# RhoA deficiency disrupts podocyte cytoskeleton and induces podocyte apoptosis by inhibiting YAP/dendrin signal

**DOI:** 10.1186/s12882-016-0287-6

**Published:** 2016-07-07

**Authors:** Zongshun Huang, Li Zhang, Yuanhan Chen, Hong Zhang, Chunping Yu, Fangjian Zhou, Zhiling Zhang, Lijuan Jiang, Ruizhao Li, Jianchao Ma, Zhuo Li, Yuxiong Lai, Ting Lin, Xinchen Zhao, Qianmei Zhang, Bin Zhang, Zhiming Ye, Shuangxin Liu, Wenjian Wang, Xinling Liang, Ruyi Liao, Wei Shi

**Affiliations:** Division of Nephrology, Guangdong General Hospital, Guangdong Academy of Medical Sciences, 106 Zhongshan No. 2 Road, Guangzhou, 510080 China; Southern Medical University, Guangzhou, Guangdong China; State Key Laboratory of Oncology in South China, Sun Yat-sen University Cancer Center, Guangzhou, Guangdong 510060 China; Department of Urology, Sun Yat-sen University Cancer Center, Guangzhou, Guangdong 510060 China

**Keywords:** RhoA, mDia, Stress fibers, YAP, Dendrin, Apoptosis

## Abstract

**Background:**

Podocyte apoptosis is a major mechanism that leads to proteinuria in many kidney diseases. However, the concert mechanisms that cause podocyte apoptosis in these kidney diseases are not fully understood. RhoA is one of Rho GTPases that has been well studied and plays a key role in regulating cytoskeletal architecture. Previous study showed that insufficient RhoA could result in rat aortic smooth muscle cell apoptosis. However, whether RhoA is involved in podocyte apoptosis remains unknown.

**Methods:**

Culture podocytes were treated with LPS, ADR or siRNA for 48 h before harvest. Subcellular immunoblotting, qRT-PCR, immunofluorescence and flow cytometry were used to exam the expression and function of RhoA or YAP in podocytes.

**Results:**

We found that the expression of RhoA and its activity were significantly decreased in LPS or ADR-injured podocytes, accompanying loss of stress fibers and increased cell apoptosis. Knocking down RhoA or its downstream effector mDia expression by siRNA also caused loss of stress fibers and podocyte apoptosis. Moreover, our results further demonstrated that RhoA deficiency could reduce the mRNA and protein expression of YAP, which had been regarded as an anti-apoptosis protein in podocyte. Silenced dendrin expression significantly abolished RhoA, mDia or YAP deficiency-induced podocyte apoptosis.

**Conclusion:**

RhoA deficiency could disrupt podocyte cytoskeleton and induce podocyte apoptosis by inhibiting YAP/dendrin signal. RhoA/mDia/YAP/dendrin signal pathway may potentially play an important role in regulating podocyte apoptosis. Maintaining necessary RhoA would be one potent way to prevent proteinuria kidney diseases.

## Background

Podocytes are highly differentiated cells with complex actin cytoskeletal architecture that play a key role in maintaining the integrity of glomerular filtration barrier, which is crucial for normal glomerular function [1–3]. Therefore, loss of podocytes will surely disturb the function of glomerular. Apoptosis is one of the main reasons that causes loss of podocyte and sequentially induces proteinuria. Accumulating evidences show that podocyte apoptosis is one of the most important mechanisms in the pathogenesis of many kidney diseases, such as chronic kidney disease [4, 5], diabetic nephropathy [6–8], focal segmental glomerular sclerosis [9, 10], et al. Thus, preventing podocyte apoptosis will be a promising therapeutic target for treating these kidney diseases. However, the concert mechanisms that cause podocyte apoptosis in these kidney diseases are still far from being fully understood.

It has been well known that severe disorders of the kidney that present with proteinuria are associated with remarkable cytoskeletal injury and foot process effacement [11]. The function of cytoskeletal architecture is mainly regulated by small GTPases belonging to the Rho GTPase family [12, 13]. Ras homolog gene family, member A (RhoA) is one of Rho GTPases that has been well studied. Active RhoA binds to its downstream effector mammalian diaphanous-related formins (mDias) to relieve mDias’ autoinhibition of the DID/DAD interaction and activates it, thereby promotes stress fibers formation [14, 15]. A baseline activity of RhoA is essential for the normal function of glomerular podoctyes, podocytes deficiency in RhoA will lead to loss of stress fibers and proteinuria [16, 17]. Accumulating evidences have showed that RhoA deficiency could result in cell apoptosis [11, 18], maybe by decreasing Yes-associated protein (YAP) expression in both A549 and HepG2 cells [19].

YAP is a major downstream cascade of the Hippo pathway which controls the expression of genes that promote cell proliferation and inhibit apoptosis [20, 21]. Under normal condition, dephosphorylated YAP localizes in the nucleus and functions as a transcriptional co-activator that mainly through interacting with TEA domain family member (TEAD) family transcriptional factors to induce target gene expression [22]. YAP phosphorylation promotes its cytoplasmic sequestration and inactivation [23, 24]. As loss of stress fibers caused by insufficient RhoA can result in reducing transcriptional activity and nuclear localization of YAP [19, 25, 26], which has been regarded as an anti-apoptosis protein in many kinds of cells [20, 27–29], including podocyte [30, 31], it is likely that RhoA deficiency may induce podocyte apoptosis by reducing nuclear YAP.

Dendrin is a PPXY motif containing dual compartment protein [32]. It was originally identified in sleep-deprived rats [33], and also detected in human kidney podocytes [34]. Ablation of the podocyte death-promoting protein dendrin can delay onset and severity of podocytes loss and proteinuria [35]. In normal podocytes, dendrin is a constituent of the glomerular slit diaphragm, where it binds to nephrin and CD2-associated protein [32]. In response to glomerular injury, dendrin relocates to the podocyte nucleus, thereby promoting cell apoptosis [36]. Previous study [30] showed that YAP can bind to dendrin in podocyte nuclei, thereby acting as an endogenous inhibitor of proapoptotic dendrin signaling in podocytes. Thus, it is possible that RhoA deficiency can induce podocyte apoptosis by inhibiting YAP/dendrin signal.

In this study, lipopolysaccharide (LPS) or adriamycin (ADR) was used to elicit podocyte cytoskeleton disruption and apoptosis, as described previously [9, 37], then we surprisingly found that RhoA expression and its activity were decreased in LPS or ADR injured podocytes. Previous studies have showed that insufficient RhoA could result in cell apoptosis [18, 19]. Thus, the aim of this study is to test whether RhoA deficiency could induce podocyte apoptosis and explore its mechanism, which may be one common reason that causes proteinuria in many kidney diseases.

## Methods

### Cell culture and treatment

The conditionally immortalized mouse podocyte cell line (MPC) was a kind gift from Dr. Jochen Reiser (Rush University Medical Center, Chicago, IL, USA), and cultured as described previously [38]. Cells were cultured at 33 °C in RPMI-1640 medium (Gibco BRL, Gaithersburg, MD, USA) supplemented with 10 % fetal bovine serum (FBS, Gibco BRL, USA) and recombinant IFN-γ (growth permissive conditions; CYT-358, ProSpec, Tany Technogene Ltd, Israel). To induce differentiation, podocytes were reseeded and cultured at 37 °C in 100 cm^2^ culture dish coated with 12 mg/ml type-I collagen (BD Bioscience, Bedford, MA, USA) and in RPMI-1640 medium supplemented with 5 % FBS, deprived of IFN-γ(growth restrictive conditions) for 10 to 13 days. After differentiation, podocytes was confirmed by the identification of synaptopodin, a podocyte differentiation marker, 10^6^cells were synchronized into quiescence by growing cells in serum-free RPMI-1640 medium for 24 h, and then treated with LPS (100 μg/ml), ADR (0.125 μg/ml) or small interfering RNA (siRNA, 50nM) for 48 h. Each reaction was repeated in triplicates.

### Transfection

The siRNAs against RhoA, mDia, YAP, dendrin and control were designed and synthesized by RIBOBIO CO., LTD. (Guangzhou, China), and were transfect into podocytes using Lipofectamine 2000 reagent (Invitrogen, Carlsbad, CA) following the manufacturer’s protocol. The sequences of siRNAs used in this study were as follows: RhoA-siRNA 5′-GGUAAGACAUGCUUGCUCA dTdT-3′, mDia-siRNA 5′-GCAAGUUCUUGUCCUCUUU dTdT-3′, YAP-siRNA 5′-CGAGAUG AGAGCACAGACA dTdT-3′, Dendrin-siRNA 5′-GGCUCCACCUUCUUAUGAA dTdT-3′.

### Immunofluorescence

Podocytes were planted on cover slides in six-well plates. After subjected to various treatments, differentiated podocytes were fixed with 4 % paraformaldehyde at room temperature for 10 min, and then treated with 0.1 % Triton X-100 for 10 min, blocked with 1 % bovine serum albumin for 20 min at room temperature, and incubated with rabbit anti-YAP (Santa Cruz, USA, 1:250) overnight at 4 °C. After incubated with the goat anti-rabbit Alexa Fluor 488 (Cell Signaling Technology, USA, 1:1000) for 1 h at room temperature, podocytes were stained with phalloidin (Cytoskeleton, USA, 1:500) for 30 min and then with DAPI (Sigma, St Louis, MO) for 5 min at room temperature. Photomicrographs were taken with laser confocal microscopy (LCSM, Zeiss KS 400, Postfach, Germany). All images were analyzed by two investigators blinded to the identity of the samples.

### Real-time quantitative-PCR

As described previously [6], RNA samples were prepared using Trizol RNA isolation system (Invitrogen, Carlsbad, CA) and reverse transcribed into cDNAs using the PrimeScriptTM RT regent Kit following the instructions provided by the manufacturer ((Takara Bio Inc, Japan), and then cDNAs were used for real-time PCR analysis by a Plantinum SYBR Green SuperMix-UDG kit (Takara Bio Inc, Japan). The primers used are listed as follows: RhoA, forward 5′-AGCTT GTGGTAAGACATGCTTG-3′, reverse 5′-GTGTCCCATAAAGCCAACTCTAC-3′, Bax, forward 5′-CTGGACCATAGGTCGGAGTG-3′, reverse 5′-AATTCGCCGGAGACACTCG-3′, GAPDH, forward 5′-AGGTCGGTGTGAACGGATTTG-3′, reverse 5′-TGTAGACCATGTAGTTGAGGT CA-3′.

### Western blotting

Whole and nuclear proteins were prepared as described previously [6]. An aliquot of cell lysates containing 30 μg of protein was separated on 10 % sodium dodecyl sulfate–polyacrylamide gels, and then transferred to polyvinylidene fluoride membranes (Amersham Biosciences). After blocked by 5 % skim milk for 1 h at room temperature, membranes were incubated overnight at 4 °C with the following primary antibodies: rabbit anti-YAP (Cell Signaling Technology, USA, 1:1000), rabbit anti-Histone (Cell Signaling Technology, USA, 1:3000), rabbit anti-RhoA (Santa Cruz, USA, 1:500), rabbit anti-Bax (Santa Cruz, USA, 1:500), rabbit anti-GAPDH (Bioworld Technology, China, 1:10000), rabbit anti-Dia (Santa Cruz, USA, 1:500), rabbit anti-Dendrin (Santa Cruz, USA, 1:500). The membranes were then incubated with anti-rabbit IgG (Jackson Immuno Research, USA, 1: 4000) at room temperature for 1 h. Finally, membranes were treated with ECL reagents (Pierce Chemical, IN, USA), followed by exposing to X-ray film (Kodak, USA). The bands of the resulting autoradiographs were quantified densitometrically using Bandscan software. Protein expression was quantified as the ratio of specific band to Histone (nuclear fractions) or GAPDH.

### Annexin V and propidium iodide staining assay

Apoptotic cells in different groups were determined using an Annexin V/PI apoptosis detection kit according to manufacturer’s protocol (Nanjing KeyGEN Biotech, China). Briefly, podocytes were resuspended with binding buffer followed by incubation with 5 ml of Annexin V (conjugated with FITC) and 5 ml of PI in the dark for 10 min. Cell fluorescence was then analyzed using a Cell Lab Quanta™ SC Flow cytometer (Beckman Colter, Inc, USA). Cells positive for Annexin V-FITC were considered as apoptosis.

### RhoA GTPase activation assay

Active RhoA was measured by G-LISA RhoA Activation Assay Biochem kit (colorimetric assay, Cytoskeleton, USA) following the manufacturer’s instruction. And the signal was measured at 490 nm with a microplate reader (MRX, Dynatech Laboratories). Total RhoA protein expression of each group was measured by WB, as described above. Results were expressed as fold activity of stimulated in relation to non-stimulated controls normalized to total RhoA protein content.

### Statistical analysis

All values are expressed as mean ± standard deviation (SD). Statistical analysis was performed using the statistical package SPSS for Windows Ver. 19.0 (SPSS, Inc., Chicago, IL, USA). Statistical analysis of the data from multiple groups was performed by analysis of variance followed by LSD or Bonferonni tests. Comparisons between two groups were conducted by Student’s *t* test. *P*-values <0.05 were considered significant.

## Results

### LPS or ADR induced podocyte apoptosis

**Fig 1:**
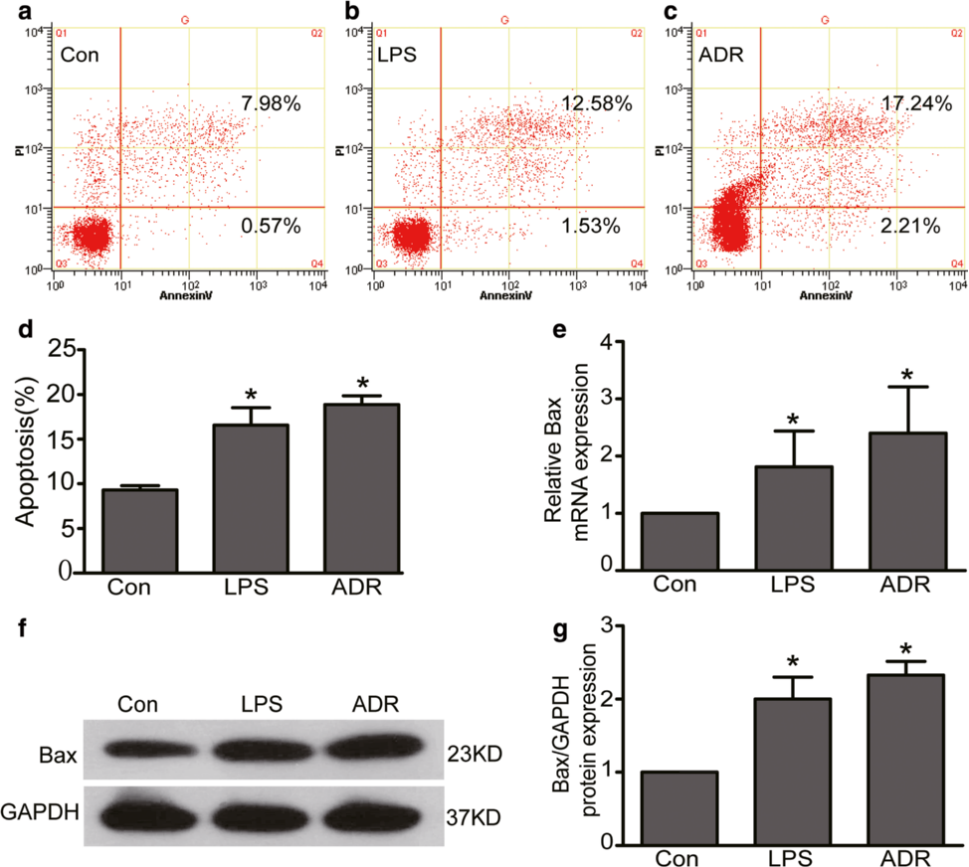
LPS or ADR induced podocyte apoptosis. **a**-**d** Podocytes were stained with Annexin V/PI for flow cytometry analysis. Cell apoptosis rate was significantly increased in LPS or ADR treated podocytes comparing to normal controls. **e** Bax mRNA expression was increased in LPS or ADR treated podocytes. **f**-**g** Bax protein expression was increased in LPS or ADR treated podocytes. Results were expressed as fold Bax of LPS or ADR group in relation to controls normalized to GAPDH. All data were from at least three independent experiments. * *P* < 0.05 vs controls

Previous studies have showed that LPS or ADR could induce podocyte apoptosis [9, 30, 37]. Thus, podocyte apoptosis model induced by LPS or ADR was used in our study. Apoptosis was verified by testing cell apoptosis rate, the mRNA and protein expression of Bax, a well recognized indicator of apoptosis [39]. As expected, results showed that cell apoptosis rates were significantly increased in LPS or ADR-injured podocytes comparing to normal controls (*P* < 0.05). Cell apoptosis rates were 9.30 ± 0.48 %, 16.58 ± 1.96 %, 18.88 ± 0.99 % in control, LPS and ADR group respectively (Fig. 1a-d). Coincided with results above, the mRNA and protein expression of Bax were also significantly increased in LPS or ADR-injured podocytes comparing to normal controls (Fig. 1e-g).

### RhoA expression and activity were decreased in LPS or ADR-injured podocytes

**Fig 2:**
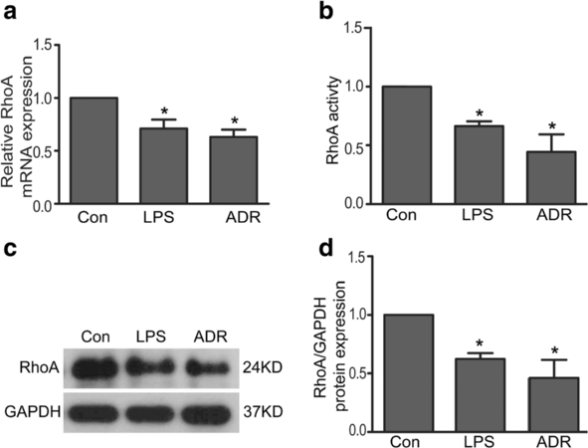
RhoA expression and activity were decreased in LPS or ADR-injured podocytes. **a** RhoA mRNA expression was decreased in LPS or ADR-injured podocytes. **b** RhoA activity was decreased in LPS or ADR injured podocytes. Results were expressed as fold activity of LPS or ADR stimulated group in relation to non-stimulated controls normalized to total RhoA protein content. **c**-**d** Total RhoA protein expression was decreased in LPS or ADR-injured podocytes. All results were expressed as fold RhoA of LPS or ADR group in relation to controls normalized to GAPDH. Data were from at least three independent experiments. * *P* < 0.05 vs controls

Since RhoA plays an important role in regulating cytoskeletal architecture, we speculate that RhoA would make response to LPS or ADR-induced podocyte cytoskeletal disruption. As shown in Fig. 2a, c-d, the mRNA and total protein expression of RhoA were significantly decreased in LPS or ADR-injured podocytes comparing to normal controls. Similarly, RhoA activity was also obviously decreased in LPS or ADR treated podocytes. The percentages of RhoA activity were 100 %, 66.35 ± 4.10 %, 44.31 ± 14.96 % in control, LPS and ADR group respectively (Fig. 2b).

### RhoA deficiency induced podocyte apoptosis

**Fig 3:**
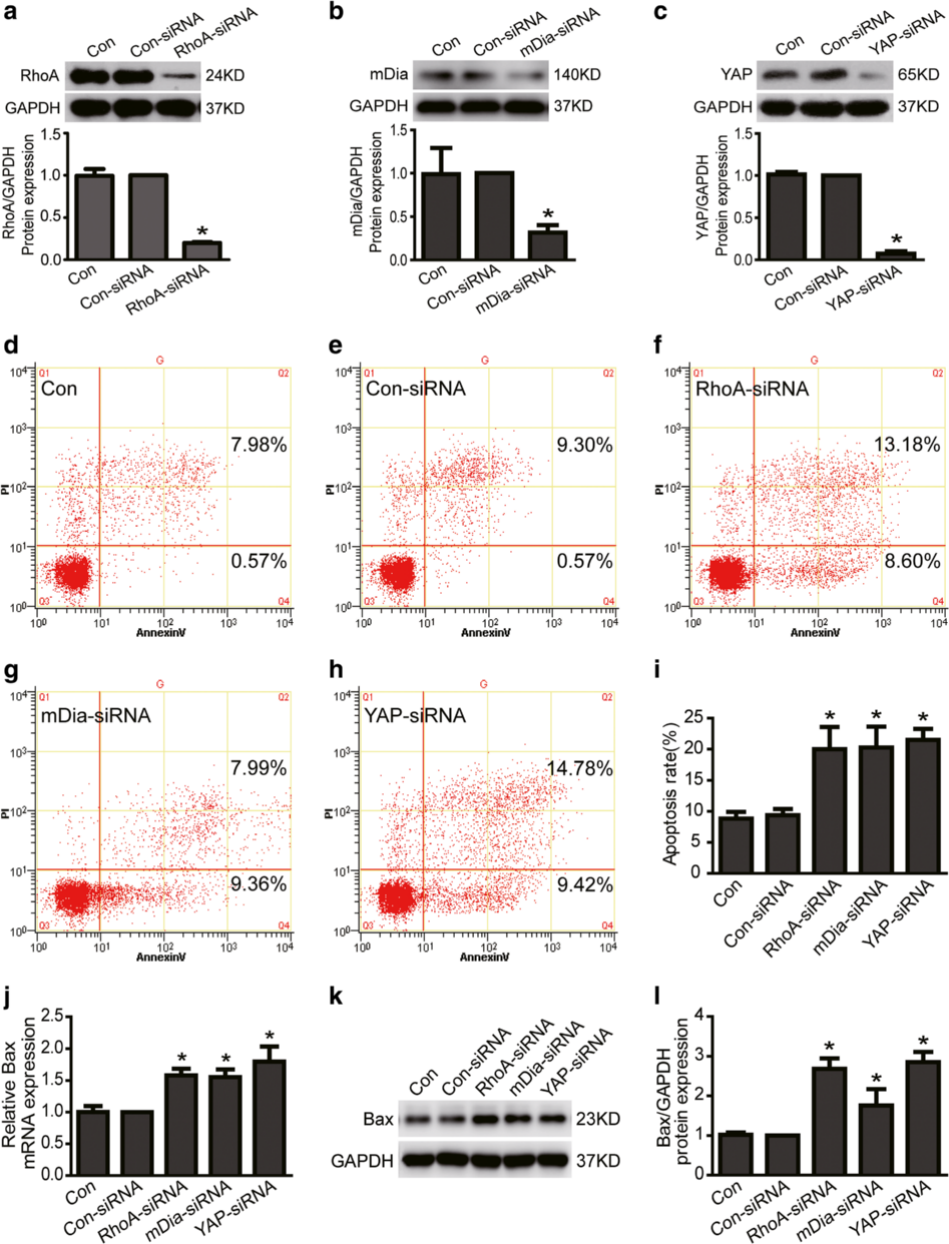
Loss of RhoA, mDia or YAP induced podocyte apoptosis. **a**-**c** Con and Con-siRNA were short for blank control and control siRNA respectively. RhoA and its downstream effector mDia and YAP protein expression was knockdown to 20 %, 31.7 % and 7.3 % respectively in siRNA treated podocytes. **d**-**i** Podocytes were stained with Annexin V/PI for flow cytometry analysis. Cell apoptosis rate was significantly increased in RhoA, mDia or YAP knockdown podocytes. **j** Bax mRNA expression was obviously increased in RhoA, mDia or YAP knockdown podocytes. **k**-**l** Bax protein expression was remarkably increased in RhoA, mDia or YAP knockdown podocytes. Results were expressed as fold Bax of RhoA-siRNA, mDia-RNA or YAP-siRNA group in relation to con-siRNA group normalized to GAPDH. There is no significant difference between control group and con-siRNA group. Data were from at least three independent experiments. * *P* < 0.05 vs con-siRNA group

Previous studies have showed that RhoA deficiency can result in A549 and HepG2 cell apoptosis [19]. To explore the role of decreased RhoA in podocytes, we studied cell apoptosis in RhoA konckdown podocytes. As shown in Fig. 3d-f, i, apoptosis was markedly increased in RhoA knockdown (to an average of 20 %, Fig. 3a) podocytes compare to siCon (*P* < 0.05). We also investigated the mRNA and protein expression of Bax, which was a well recognized indicator of apoptosis. As shown in Fig. 3j-l, the mRNA and protein expression of Bax were significantly increased in RhoA silenced podocytes. These data strongly indicated that RhoA deficiency could induce podocyte apoptosis.

### YAP expression was reduced in LPS or ADR-injured podocytes

**Fig 4:**
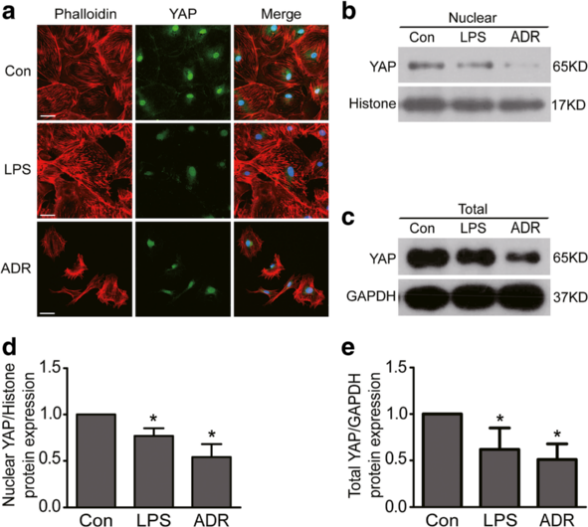
YAP expression was reduced in LPS or ADR-injured podocytes. **a** Confocal images of podocytes showing expression of YAP (green), Phalloidin-stained stress fiber (red) and DAPI-stained nuclei (blue). Compared to controls, nuclear and cytoplasmic YAP and stress fiber were reduced in LPS or ADR-injured podocytes. Scale bar = 50 μm. **b** & **d** WB results show that nuclear YAP protein expression was reduced in both LPS and ADR treated podocytes. **c** & **e** WB results show that total YAP protein expression was reduced in both LPS and ADR treated podocytes. All results were expressed as fold YAP of LPS or ADR group in relation to controls normalized to Histone or GAPDH. Data were from at least three independent experiments. * *P* < 0.05 vs controls

YAP has been regarded as an anti-apoptosis protein in podocyte [30]. Active YAP localizes in the nucleus and functions as a transcriptional co-activator to induce target gene expression [22]. To further explore the underlying mechanism of RhoA deficiency-induced podocyte apoptosis, we next investigated the expression of transcriptional co-activator YAP in LPS or ADR-injured podocytes. We used confocal miscroscopy to examine the localization of YAP. Results showed that both nuclear and cytoplasmic YAP were obviously reduced in LPS or ADR treated podocytes comparing to normal controls (Fig. 4a). Results from Immunoblotting also showed that both nuclear and total YAP were reduced in LPS or ADR treated podocytes (Fig. 4b-e). These data indicated that YAP protein expression was significantly decreased in LPS or ADR-injured podocytes.

### Loss of mDia or YAP induced podocyte apoptosis

Since lost of RhoA induced podocyte apoptosis, we investigated whether loss of its downstream effector mDia or YAP could also induce podocyte apoptosis. Here, we used siRNA to knock down mDia (to an average of 31.7 %, Fig. 3b) or YAP (to an average of 7.3 %, Fig. 3c) expression in podocytes. As shown in Fig. 3d-i, podocyte apoptosis rates analyzed by flow cytometer were 8.83 ± 1.07 %, 9.36 ± 1.02 %, 20.26 ± 3.37 %, 21.50 ± 1.76 % in control, control-siRNA, mDia-siRNA and YAP-siRNA group respectively. Cell apoptosis rate was significantly increased in mDia or YAP knockdown podocytes (*P* < 0.05). In addition, we further found that the mRNA and protein expression of Bax were also significantly increased in mDia or YAP knockdown podocytes (Fig. 3j-l). Thus, these data strongly indicated that mDia or YAP deficiency could induce podocyte apoptosis.

### Loss of RhoA/mDia decreased YAP expression in podocytes

**Fig 5:**
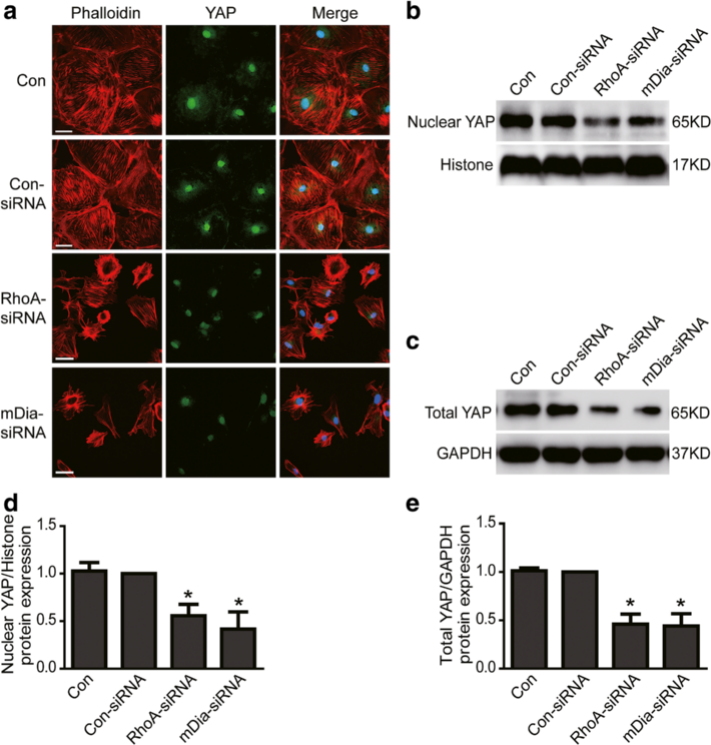
YAP expression was reduced in RhoA or mDia knockdown podocytes. **a** Con and Con-siRNA were short for blank control and control siRNA respectively. Confocal images of podocytes showing expression of YAP (green), Phalloidin-stained stress fiber (red) and DAPI-stained nuclei (blue). Compared to con-siRNA, nuclear and cytoplasmic YAP and stress fiber were obviously reduced in RhoA-siRNA or mDia-RNA treated podocytes. Scale bar = 50 μm. **b** & **d** WB results show that nuclear YAP protein expression was reduced in RhoA-siRNA or mDia-RNA treated podocytes. **c** & **e** Total YAP protein expression was reduced in RhoA-siRNA or mDia-RNA treated podocytes. All results were expressed as fold YAP of RhoA-siRNA or mDia-RNA group in relation to con-siRNA group normalized to Histone or GAPDH. There is no significant difference between control group and con-siRNA group. Data were from at least three independent experiments. * *P* < 0.05 vs con-siRNA group

To further explore the underlying mechanism of RhoA deficiency-induced podocyte apoptosis, we investigated the expression of YAP in RhoA or its downstream effector mDia knockdown podocytes. As shown in Fig. 5a, results from immunofluorescence demonstrated that both nuclear and cytoplasmic YAP protein expression were obviously decreased in RhoA or mDia knockdown podocytes comparing to con-siRNA treated podocytes. Similar results from western blotting to analyze the protein expression of YAP were also obtained in RhoA or mDia knockdown podocytes (Fig. 5b-e). These data indicated that RhoA/mDia could positively regulate YAP expression.

In addition, we investigated the expression of Phalloidin-stained stress fiber, which had been regarded as a regulator of YAP [25, 26, 30], in RhoA or mDia silenced podocytes. As shown in Fig. 5a, Phalloidin-stained stress fibers were obviously decreased in RhoA or mDia silenced podocytes. This is consistent with above results that stress fibers were also decreased (Fig. 4a) in LPS or ADR-injured podocytes. These data indicated that RhoA/mDia may regulate YAP expression by stress fiber.

### Dendrin mediated RhoA/mDia/YAP deficiency-induced podocyte apoptosis

**Fig 6:**
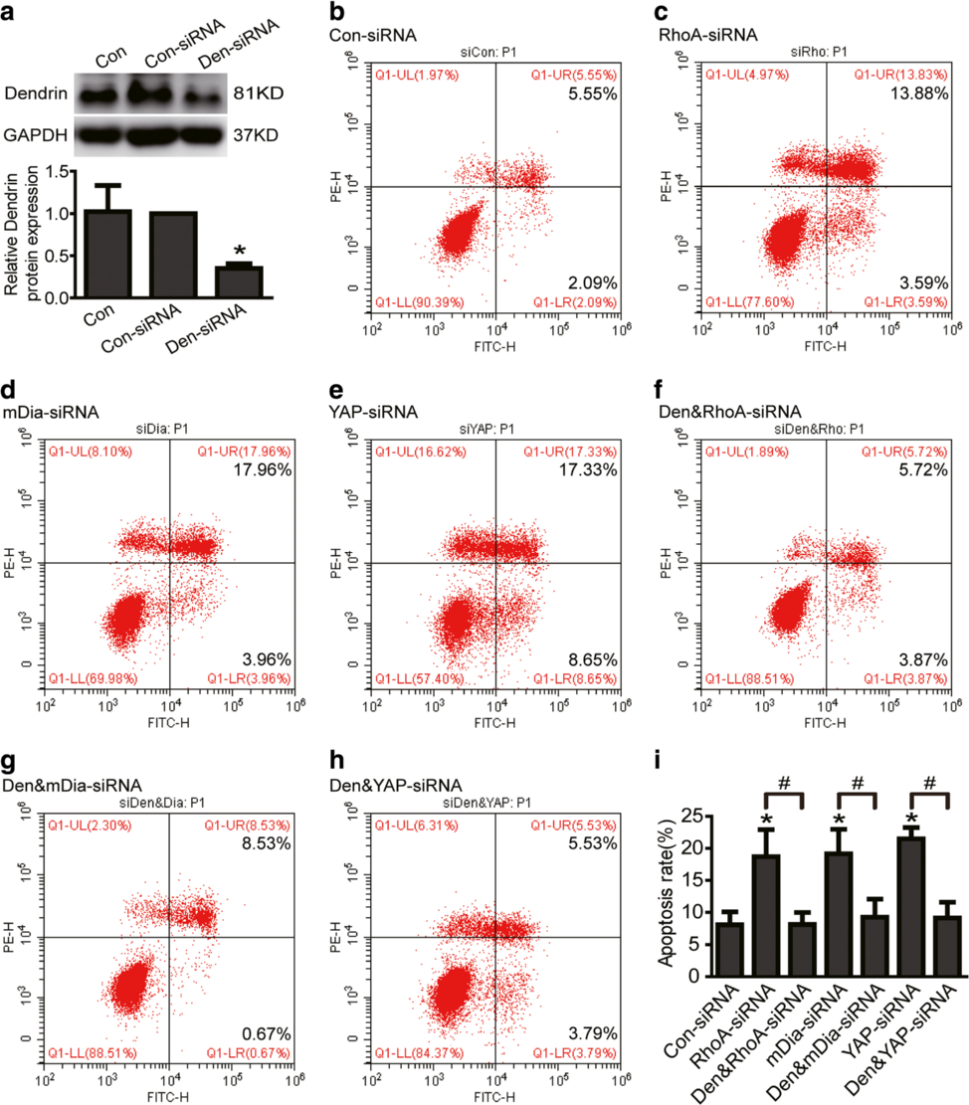
Dendrin mediated RhoA/mDia/YAP deficiency-induced podocyte apoptosis. **a** Dendrin protein expression was knockdown to about 35 % in Den-siRNA treated podocytes. **b**-**i** Con-siRNA, Den&RhoA-siRNA, Den&mDia-siRNA, Den&YAP-siRNA were short for control siRNA, Dendrin-siRNA plus RhoA-siRNA, Dendrin-siRNA plus mDia-siRNA, Dendrin-siRNA plus YAP-siRNA respectively. Podocytes were stained with Annexin V/PI for flow cytometry analysis. Cell apoptosis rate was obviously increased in RhoA, mDia or YAP knockdown podocytes. Knocking down dendrin expression with siRNA significantly abolished RhoA, mDia or YAP deficiency-induced podocyte apoptosis. All data were from at least three independent experiments. * *P* < 0.05 vs con-siRNA group. # *P* < 0.05

Previous study had showed that YAP could bind to dendrin in podocyte nuclei and inhibit dendrin-mediated podocyte apoptosis [30]. Thus, to further explore the underlying mechanism of RhoA/mDia deficientcy-induced podocyte apoptosis, we investigated whether silenced the proapoptotic dendrin signaling could block RhoA/mDia deficientcy-induced podocyte apoptosis. As shown in Fig. 6, dendrin protein expression was knockdown to about 35 % in Den-siRNA treated podocytes (Fig. 6a), podocyte apoptosis rates analyzed by flow cytometer were 8.09 ± 2.00 %, 18.69 ± 4.24 %, 19.15 ± 3.84 %, 21.50 ± 1.76 %, 8.15 ± 1.83 %, 9.26 ± 2.84 %, 9.13 ± 2.46 % in control siRNA, RhoA-siRNA, mDia-siRNA, YAP-siRNA, Dendrin-siRNA plus RhoA-siRNA, Dendrin-siRNA plus mDia-siRNA, Dendrin-siRNA plus YAP-siRNA group respectively (Fig. 6b-i). Cell apoptosis rate was obviously increased in RhoA, mDia or YAP knockdown podocytes, however, knocking down dendrin expression with siRNA significantly abolished RhoA/mDia/YAP deficiency-induced podocyte apoptosis. Therefore, these data demonstrated that dendrin mediated RhoA/mDia/YAP deficiency-induced podocyte apoptosis.

## Discussion

Podocytes are very special cells with complex actin cytoskeletal architecture that play a pivotal role in maintaining normal glomerular function [1–3]. Loss of podocyte caused by apoptosis is commonly happened in the pathogenesis of many kidney diseases, such as chronic kidney disease [4, 5], diabetic nephropathy [6–8], focal segmental glomerular sclerosis [9, 10], et al. However, the mechanism mediating podocyte apoptosis remains incompletely understood. Therefore, exploring the mechanisms that cause podocyte apoptosis in these kidney diseases is imperative.

**Fig 7:**
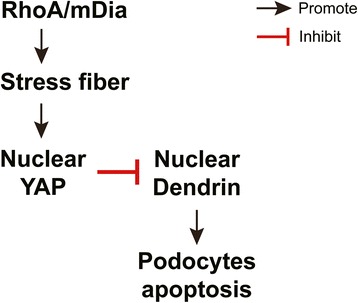
The proposed mechanism of RhoA deficiency-induced podocyte apoptosis. Black arrows indicate positive regulation, whereas red lines indicate negative regulation

In this study, LPS or ADR was used to induce podocyte cytoskeleton disruption and apoptosis. We found that RhoA expression and its activity were significantly decreased in LPS or ADR-injured podocytes. Moreover, we found podocyte apoptosis was increased in RhoA knockdown podocytes. We also further demonstrated that RhoA deficiency could reduce the mRNA and protein expression of YAP, which had been regarded as an anti-apoptosis protein in podocyte. Knocking down dendrin expression with siRNA significantly abolished RhoA, mDia or YAP deficiency-induced podocyte apoptosis. Collectively, these data indicated that RhoA/mDia/YAP/dendrin signal pathway may potentially play an important role in regulating podocyte apoptosis (Fig. 7).

RhoA is one of Rho GTPases that has been well studied and regarded as a main regulator of the function of cytoskeletal architecture [12, 13]. A baseline activity of RhoA is essential for the normal function of glomerular podoctyes, and insufficient RhoA leads to loss of stress fibers, foot process effacement and proteinuria. In a traditionally view, a stationary podocyte phenotype reflects a stable foot process structure with intact slit diaphragms, whereas hypermotility manifests as foot process effacement in vivo. Stimuli that lead to foot process effacement commonly cause hypermotility of cultured podocytes [40] and proteinuria in glomerular diseases [16]. Therefore, RhoA can prevent proteinuria by stabilizing the podocyte foot process structure, whereas inactivation of RhoA will cause hypermotility and sequentially induces proteinuria. However, in our study, we demonstrated that insufficient RhoA or its downstream effector mDia [14] could cause podocyte apoptosis, which was another main reason that would cause proteinuria. These data indicate that podocyte apoptosis is probably a new mechanism of proteinuria induced by RhoA deficiency.

YAP is a major downstream cascade of the Hippo pathway and dephosphorylated YAP localizes in the nucleus and functions as a transcriptional co-activator to induce target gene expression in normal condition [22]. It has a well described role in controlling the expression of genes that promote cell proliferation and inhibit apoptosis [20, 21]. Evidence had showed that YAP could also serve as a physiologic inhibitor of podocyte apoptosis [30]. This was consistent with our results that apoptosis was increased in YAP knockdown podocytes. Our data further demonstrated that YAP expression and its activity were decreased in RhoA or it downstream effector mDia knockdown podocytes. Similar results had also been obtained in A549 cells, HepG2 cells [19] and mesenchymal stem cells [25] from previous studies. Therefore, these data indicate that YAP may mediate RhoA/mDia deficiency-induced podocyte apoptosis.

To further explore the underlying mechanism of RhoA/mDia deficiency-induced apoptosis, we studied how RhoA regulate YAP. Previous studies had showed that RhoA could work through actin cytoskeleton to promote stress fibers formation, thereby regulated YAP phosphorylation in Lats1/2 kinase activity dependent [19, 41–43] or independent [25, 44, 45] pathway. Similarly, in our study we found that podocyte deficiency in RhoA or mDia could significantly lead to loss of stress fibers, thereby decreased YAP expression to induce podocyte apoptosis. Therefore, RhoA/mDia/stress fibers/YAP may be an important signal pathway to regulate podocyte apoptosis. And insufficient RhoA/mDia/stress fibers/YAP pathway activity in podocyte may be one common reason that causes proteinuria in many kidney diseases.

Since YAP is known to inhibit dendrin mediated apoptosis in podocytes [30], to define if RhoA activation of YAP could inhibit apoptosis through dendrin, and to strengthen the connection among RhoA, mDia, YAP and dendrin in podocytes, we silenced dendrin in RhoA, mDia or YAP knockdown podocytes and studied if cell apoptosis could be prevented. As expected, knocking down dendrin expression with siRNA significantly abolished RhoA, mDia or YAP deficiency-induced podocyte apoptosis. Thus, these data demonstrate that dendrin mediates RhoA/mDia/YAP deficiency-induced podocyte apoptosis.

Taken together, the present study demonstrated that RhoA expression and its activity were significantly decreased while cell apoptosis was increased in LPS or ADR-injured podocytes. Knocked down RhoA and its downstream effector mDia expression induced podocyte apoptosis. Moreover, RhoA deficiency-induced podocyte apoptosis was probably mediated by reducing YAP expression, silenced dendrin expression significantly abolished RhoA, mDia or YAP deficiency-induced podocyte apoptosis. Collectively, our data indicate that RhoA/mDia/YAP/dendrin signal pathway may potentially play an important role in regulating podocyte apoptosis.

## Conclusions

RhoA/mDia/YAP/dendrin signal pathway may potentially play an important role in regulating podocyte apoptosis. It may be one common reason that causes proteinuria in many kidney diseases. Maintaining necessary RhoA would be one potent way to prevent proteinuria kidney diseases.

## Abbreviations

ADR, Adriamycin; LPS, Lipopolysaccharide; mDia, Mammalian diaphanous-related formin; RhoA, Ras homolog gene family, member A; siRNA, Small interfering RNA; YAP, Yes-associated protein

## References

[1] Faul C, Donnelly M, Merscher-Gomez S, Chang YH, Franz S, Delfgaauw J, Chang JM, Choi HY, Campbell KN, Kim K (2008). The actin cytoskeleton of kidney podocytes is a direct target of the antiproteinuric effect of cyclosporine A. Nat Med.

[2] Wei C, Moller CC, Altintas MM, Li J, Schwarz K, Zacchigna S, Xie L, Henger A, Schmid H, Rastaldi MP (2008). Modification of kidney barrier function by the urokinase receptor. Nat Med.

[3] Kerjaschki D (2001). Caught flat-footed: podocyte damage and the molecular bases of focal glomerulosclerosis. J Clin Invest.

[4] Canaud G, Bienaime F, Viau A, Treins C, Baron W, Nguyen C, Burtin M, Berissi S, Giannakakis K, Muda AO (2013). AKT2 is essential to maintain podocyte viability and function during chronic kidney disease. Nat Med.

[5] Yamamoto Y, Yamamoto H (2012). Interaction of receptor for advanced glycation end products with advanced oxidation protein products induces podocyte injury. Kidney Int.

[6] Li R, Zhang L, Shi W, Zhang B, Liang X, Liu S, Wang W (2013). NFAT2 mediates high glucose-induced glomerular podocyte apoptosis through increased Bax expression. Exp Cell Res.

[7] Susztak K, Raff AC, Schiffer M, Bottinger EP (2006). Glucose-induced reactive oxygen species cause apoptosis of podocytes and podocyte depletion at the onset of diabetic nephropathy. Diabetes.

[8] Fiorina P, Vergani A, Bassi R, Niewczas MA, Altintas MM, Pezzolesi MG, D'Addio F, Chin M, Tezza S, Ben Nasr M, et al. Role of podocyte B7-1 in diabetic nephropathy. J Am Soc Nephrol. 2014;25(7):1415–29. 10.1681/ASN.2013050518 PMC4073425 24676639

[9] Marshall CB, Krofft RD, Pippin JW, Shankland SJ (2010). CDK inhibitor p21 is prosurvival in adriamycin-induced podocyte injury, in vitro and in vivo. Am J Physiology Renal Physiol.

[10] Daehn I, Casalena G, Zhang T, Shi S, Fenninger F, Barasch N, Yu L, D'Agati V, Schlondorff D, Kriz W, et al. Endothelial mitochondrial oxidative stress determines podocyte depletion in segmental glomerulosclerosis. J Clin Invest. 2014;124(4):1608–21. 10.1172/JCI71195 PMC3973074 24590287

[11] Jeruschke S, Buscher AK, Oh J, Saleem MA, Hoyer PF, Weber S, Nalbant P (2013). Protective effects of the mTOR inhibitor everolimus on cytoskeletal injury in human podocytes are mediated by RhoA signaling. PLoS One.

[12] Asanuma K, Yanagida-Asanuma E, Faul C, Tomino Y, Kim K, Mundel P (2006). Synaptopodin orchestrates actin organization and cell motility via regulation of RhoA signalling. Nat Cell Biol.

[13] Yanagida-Asanuma E, Asanuma K, Kim K, Donnelly M, Young Choi H, Hyung Chang J, Suetsugu S, Tomino Y, Takenawa T, Faul C (2007). Synaptopodin protects against proteinuria by disrupting Cdc42:IRSp53:Mena signaling complexes in kidney podocytes. Am J Pathol.

[14] Sun H, Schlondorff JS, Brown EJ, Higgs HN, Pollak MR (2011). Rho activation of mDia formins is modulated by an interaction with inverted formin 2 (INF2). Proc Natl Acad Sci U S A.

[15] Sun H, Schlondorff J, Higgs HN, Pollak MR (2013). Inverted formin 2 regulates actin dynamics by antagonizing Rho/diaphanous-related formin signaling. J Am Soc Nephrol.

[16] Kistler AD, Altintas MM, Reiser J (2012). Podocyte GTPases regulate kidney filter dynamics. Kidney Int.

[17] Buvall L, Rashmi P, Lopez-Rivera E, Andreeva S, Weins A, Wallentin H, Greka A, Mundel P (2013). Proteasomal degradation of Nck1 but not Nck2 regulates RhoA activation and actin dynamics. Nat Commun.

[18] Bobak D, Moorman J, Guanzon A, Gilmer L, Hahn C (1997). Inactivation of the small GTPase Rho disrupts cellular attachment and induces adhesion-dependent and adhesion-independent apoptosis. Oncogene.

[19] Zhang Y, Xia H, Ge X, Chen Q, Yuan D, Leng W, Chen L, Tang Q, Bi F (2014). CD44 acts through RhoA to regulate YAP signaling. Cell Signal.

[20] Huang J, Wu S, Barrera J, Matthews K, Pan D (2005). The hippo signaling pathway coordinately regulates cell proliferation and apoptosis by inactivating yorkie, the drosophila homolog of YAP. Cell.

[21] Pan D (2010). The hippo signaling pathway in development and cancer. Dev Cell.

[22] Cao X, Pfaff SL, Gage FH (2008). YAP regulates neural progenitor cell number via the TEA domain transcription factor. Genes Dev.

[23] Hao Y, Chun A, Cheung K, Rashidi B, Yang X (2008). Tumor suppressor LATS1 is a negative regulator of oncogene YAP. J Biol Chem.

[24] Zhao B, Wei X, Li W, Udan RS, Yang Q, Kim J, Xie J, Ikenoue T, Yu J, Li L (2007). Inactivation of YAP oncoprotein by the Hippo pathway is involved in cell contact inhibition and tissue growth control. Genes Dev.

[25] Dupont S, Morsut L, Aragona M, Enzo E, Giulitti S, Cordenonsi M, Zanconato F, Le Digabel J, Forcato M, Bicciato S (2011). Role of YAP/TAZ in mechanotransduction. Nature.

[26] Reginensi A, Scott RP, Gregorieff A, Bagherie-Lachidan M, Chung C, Lim DS, Pawson T, Wrana J, McNeill H (2013). Yap- and Cdc42-dependent nephrogenesis and morphogenesis during mouse kidney development. PLoS Genet.

[27] Yu FX, Zhao B, Panupinthu N, Jewell JL, Lian I, Wang LH, Zhao J, Yuan H, Tumaneng K, Li H (2012). Regulation of the Hippo-YAP pathway by G-protein-coupled receptor signaling. Cell.

[28] Camargo FD, Gokhale S, Johnnidis JB, Fu D, Bell GW, Jaenisch R, Brummelkamp TR (2007). YAP1 increases organ size and expands undifferentiated progenitor cells. Curr Biol.

[29] Dong J, Feldmann G, Huang J, Wu S, Zhang N, Comerford SA, Gayyed MF, Anders RA, Maitra A, Pan D (2007). Elucidation of a universal size-control mechanism in drosophila and mammals. Cell.

[30] Campbell KN, Wong JS, Gupta R, Asanuma K, Sudol M, He JC, Mundel P (2013). Yes-associated protein (YAP) promotes cell survival by inhibiting proapoptotic dendrin signaling. J Biol Chem.

[31] Schwartzman M, Reginensi A, Wong JS, Basgen JM, Meliambro K, Nicholas SB, D'Agati V, McNeill H, Campbell KN. Podocyte-specific deletion of Yes-associated protein causes FSGS and progressive renal failure. J Am Soc Nephrol. 2015;27:216–26. 10.1681/ASN.2014090916 PMC4696566 26015453

[32] Asanuma K, Campbell KN, Kim K, Faul C, Mundel P (2007). Nuclear relocation of the nephrin and CD2AP-binding protein dendrin promotes apoptosis of podocytes. Proc Natl Acad Sci U S A.

[33] Neuner-Jehle M, Denizot JP, Borbely AA, Mallet J (1996). Characterization and sleep deprivation-induced expression modulation of dendrin, a novel dendritic protein in rat brain neurons. J Neurosci Res.

[34] Duner F, Patrakka J, Xiao Z, Larsson J, Vlamis-Gardikas A, Pettersson E, Tryggvason K, Hultenby K, Wernerson A (2008). Dendrin expression in glomerulogenesis and in human minimal change nephrotic syndrome. Nephrol Dial Transplant.

[35] Weins A, Wong JS, Basgen JM, Gupta R, Daehn I, Casagrande L, Lessman D, Schwartzman M, Meliambro K, Patrakka J (2015). Dendrin ablation prolongs life span by delaying kidney failure. Am J Pathol.

[36] Kodama F, Asanuma K, Takagi M, Hidaka T, Asanuma E, Fukuda H, Seki T, Takeda Y, Hosoe-Nagai Y, Asao R (2013). Translocation of dendrin to the podocyte nucleus in acute glomerular injury in patients with IgA nephropathy. Nephrol Dial Transplant.

[37] Bao H, Ge Y, Peng A, Gong R (2015). Fine-tuning of NFkappaB by glycogen synthase kinase 3beta directs the fate of glomerular podocytes upon injury. Kidney Int.

[38] Mundel P, Reiser J, Zuniga Mejia Borja A, Pavenstadt H, Davidson GR, Kriz W, Zeller R (1997). Rearrangements of the cytoskeleton and cell contacts induce process formation during differentiation of conditionally immortalized mouse podocyte cell lines. Exp Cell Res.

[39] Reed JC (2006). Proapoptotic multidomain Bcl-2/Bax-family proteins: mechanisms, physiological roles, and therapeutic opportunities. Cell Death Differ.

[40] Mundel P, Reiser J (2010). Proteinuria: an enzymatic disease of the podocyte?. Kidney Int.

[41] Sansores-Garcia L, Bossuyt W, Wada K, Yonemura S, Tao C, Sasaki H, Halder G (2011). Modulating F-actin organization induces organ growth by affecting the hippo pathway. EMBO J.

[42] Rauskolb C, Pan G, Reddy BV, Oh H, Irvine KD (2011). Zyxin links fat signaling to the hippo pathway. PLoS Biol.

[43] Wada K, Itoga K, Okano T, Yonemura S, Sasaki H (2011). Hippo pathway regulation by cell morphology and stress fibers. Development.

[44] Halder G, Dupont S, Piccolo S (2012). Transduction of mechanical and cytoskeletal cues by YAP and TAZ. Nat Rev Mol Cell Biol.

[45] Mammoto T, Ingber DE (2010). Mechanical control of tissue and organ development. Development.

